# Identification of Volatile Molecules and Bioactivity of Gruyt Craft Beer Enriched with *Citrus aurantium* var. *dulcis* L. Essential Oil

**DOI:** 10.3390/ijms25010350

**Published:** 2023-12-26

**Authors:** Cosimo Taiti, Antonella Di Sotto, Giovanni Stefano, Ester Percaccio, Matteo Iannone, Andrea Marianelli, Stefania Garzoli

**Affiliations:** 1 Department of Agriculture, Food, Environmental and Forest, Florence University, Sesto Fiorentino, 50019 Firenze, Italy; cosimo.taiti@unifi.it; 2 Department of Physiology and Pharmacology “V. Erspamer”, Sapienza University of Rome, 00185 Rome, Italy; antonella.disotto@uniroma1.it (A.D.S.); ester.percaccio@uniroma1.it (E.P.); 3 Department of Biology, Florence University, 50121 Firenze, Italy; giovanni.stefano@unifi.it; 4 Circolo ARCI La Staffetta, 56011 Calci, Italy; arcilastaffetta@gmail.com (M.I.); andreamarianelli93@gmail.com (A.M.); 5 Department of Chemistry and Technology of Drug, Sapienza University, 00185 Rome, Italy

**Keywords:** chemical composition, HS-SPME, GC-MS, PTR-ToF-MS, antioxidant activity, chelating activity, antiglycative activity, cytoprotection, reactive oxygen species

## Abstract

In this work, for the first time, a gruyt beer and the same one after the addition of *Citrus aurantium* essential oil (AEO), were investigated to determine the composition of the volatile fraction. The applied analytical techniques, such as Head Space/Solid Phase Microextraction-Gas Chromatography-Mass Spectrometry (HS/SPME-GC-MS) and Proton Transfer Reaction-Time of Flight-Mass Spectrometer (PTR-ToF-MS), allowed us to identify the content of volatile organic compounds (VOCs). From the comparison between the two beer samples, it showed that the one after the addition of AEO was particularly richened in limonene and a series of minor terpene compounds. AEO was also characterized by GC/MS analysis and the results showed that limonene reached 95%. Confocal microscopy was used to look at riboflavin autofluorescence in yeast cells. It was found that beer with AEO had twice as much fluorescence intensity as the control. A spectrophotometric analysis of total polyphenols, tannins, and flavonoids, and a bioactivity screening, including 2,2-diphenyl-1-picrylhydrazyl (DPPH) and 2,2′-Azinobis-(3-Ethylbenzthiazolin-6-Sulfonic Acid) (ABTS) radical scavenger, chelating, reducing, antiglycative ones, were also carried out. Moreover, the tolerability of the tested samples in human H69 cholangiocytes and the cytoprotection towards the tert-butyl hydroperoxide (tBOOH)-induced oxidative damage were evaluated. Under our experimental conditions, the beers were found to be able to scavenge DPPH and ABTS radicals and chelate iron ions, despite weak antiglycative and reducing properties. The tested samples did not affect the viability of H69 cholangiocytes up to the highest concentrations; moreover, no signs of cytoprotection against the damage induced by tBOOH were highlighted. Adding AEO to beer resulted in a moderate enhancement of its DPPH scavenging and chelating abilities, without improvements in the other assays. Conversely, AEO and its major compound limonene were ineffective when assessed at the concentrations added to beer. This evidence suggests that the addition of AEO may enhance the organoleptic features of the beer and slightly potentiate some of its bioactivities.

## 1. Introduction

Beer is an alcoholic drink produced by extracting raw materials such as yeast, malt, and hops with water followed by boiling and fermentation. The production of beer involves several steps with the aim of converting wheat starches into sugar, extracting the sugar with water, and then fermenting it with yeast to produce this alcoholic and slightly carbonated drink.

The strength of beer can be measured by the percentage by volume of ethyl alcohol. Strong beers exceed 4% while light beers are fully fermented low-carb beers where enzymes are used to convert normally unfermentable (and high-calorie) carbohydrates into fermentable form. In low-alcohol beers (0.5 to 2.0% alcohol) and “non-alcoholic” beers (less than 0.1% alcohol), the alcohol is removed after fermentation by low-pressure vacuum evaporation temperatures or membrane filtration.

Different varieties of hops (*Humulus lupulus*, Carl Linnaeus, 1753) are selected and bred for the bitter and aromatic qualities they impart to beer. The female flowers, or cones, produce tiny glands that contain chemicals useful in brewing. Humulons are the chemical constituents extracted during the boiling of the wort. A fraction of these, the α-acids, are isomerized by heat to form the related iso-α-acids, responsible for the characteristic bitter taste of beer.

Apart from these, beer also contains a number of volatile compounds closely related to the taste, aroma, and flavor of the drink. This fraction includes countless components belonging to different chemical classes, including higher alcohols, esters, fatty acids, carbonyl compounds, sulfur compounds, and furans [1].

Since the 1990s, there has been a surge in microbreweries in the United States, fueling a new craft beer market. The use of craft beer continues to grow and with this expansion comes new styles and flavors [2]. The word Gruyt (or Grut or Gruit) derives from German and originally indicated a mixture of herbs and spices according to medieval traditions. Gruyt is native to the Netherlands, Belgium, and parts of Germany. The herb mix is not fixed, but most use heather, ivy, horehound, mugwort, and yarrow. Other ingredients could include juniper berries, ginger, cumin seeds, nutmeg, and cinnamon. As soon as hops were “discovered”, the use of herbs took a nosedive. Luckily, with the recent craft beer craze, brewers around the world have tried their hand at brewing beer using herbs. Currently, gruyt indicates a type of beer that involves the use of herbs to season the beer to give it a characteristic aroma with bitter notes and also to prolong its shelf life. Each brewery has its own secret recipe including different herbs and occasionally even hops in varying proportions to produce unique flavors and effects.

Essential oils (EOs) are complex mixtures of low molecular weight compounds extracted from plants by distillation processes. Their constituents are mainly terpenoids and phenylpropanoids which are also responsible for the biological properties of EOs. They are commonly used for various therapeutic purposes, for applications in the pharmaceutical and cosmetology fields, and also as flavoring agents in food and drink products [3,4,5]. In the food industry, thanks to their antimicrobial properties against food-borne pathogens in particular [6], the EOs are used for preservative purposes as they are considered a valid substitute for synthetic food additives due to the increase in shelf-life of the product [7]. In addition, EOs also act as a natural flavoring agent thanks to their high terpenoid content [8].

*Citrus* is a genus belonging to the Rutaceae family and includes important crops such as oranges, lemons, limes, etc. *Citrus* fruits are widely used in cooking as a condiment and as an integral part in the preparation of desserts. Among EOs, those obtained from citrus fruits (CEOs), have found greater application in the food sector both for their aroma and flavor and for their marked broad-spectrum insecticidal, antibacterial, and antifungal properties [9,10]. Furthermore, CEOs are also used for food safety as constituents of food storage packaging [11].

Over time, increasing consumer expectations have led to many technological challenges in the beer production process which in turn have led to new preparation methods for different types of beer. In this work, for the first time, a gruyt craft beer prepared with the addition of *Citrus aurantium* var. *dulcis* essential oil (AEO), was investigated in order to evaluate the effects on the volatile aromatic profile of the beer. For this purpose, two complementary analytical techniques, such as Head Space-Solid Phase Microextraction-Gas Chromatography/Mass Spectrometry (HS-SPME-GC/MS) and Proton-Transfer-Reaction Time-of-Flight Mass Spectrometer (PTR-ToF-MS), were applied to better describe the content of volatile organic compounds (VOCs) emitted from gruyt beer before and after the addition of AEO. GC/MS analysis was carried out to characterize the chemical composition of AEO.

Furthermore, the samples of beer were evaluated for the total content of polyphenols, tannins, and flavonoids, and for their antioxidant properties. Particularly, the radical scavenger activity towards DPPH and ABTS radicals, iron chelating and reducing abilities, and antiglycative activities were assessed. Moreover, the possible cytoprotection against the oxidative damage induced by tert-butyl hydroperoxide (tBOOH) in human H69 cholangiocytes was evaluated. H69 cell was chosen as a model of the gastro-intestinal tract in order to predict the possible benefits of the tested samples against food toxicants. Along with the beer samples, AEO and limonene, its major compound, were tested in order to evaluate the possible benefits arising from adding AEO to beer.

## 2. Results

### 2.1. SPME-GC/MS Chemical Composition of Beers

The analyses conducted via SPM-GC/MS allowed for the identification of twenty-one volatile organic compounds (VOCs) listed in Table 1.

The control beer was characterized by the presence of alcohol and ester compounds. Among the former, active amyl alcohol (17.0%) was prominent, while in the latter, ethyl caprylate prevailed (47.6%). The only monoterpene found was limonene (2.0%).

In addition to the VOCs that were found in the control beer, in the beer with AEO, a group of terpene compounds closely linked to the added oil were found. By far the most significant component was limonene which covered a percentage of 96.6%. Among the detected monoterpenes, *β*-myrcene was the most abundant (1.3%), followed by *α*-pinene (0.3%), terpinolene (0.2%), and *γ*-terpinene (0.1%). All other monoterpenes were present in trace amounts. The only two sesquiterpenes that exceeded the 0.1% threshold were *β*-caryophyllene (0.2%) and *β*-eudesmene (0.4%).

### 2.2. PTR-ToF-MS: Determination of Volatile Compounds from Beers

The first aim was to understand if the addition of AEO changed the beer aroma profile. All the compounds detected with an average intensity emission >1 ppbv were reported in Table 2. It emerges how the control beer (without AEO) showed a less complex aromatic profile than the beer with AEO, which is instead characterized by a higher (1) number of VOCs; (2) total emission of VOCs; (3) emission of terpene compounds (Table 2). In particular, the total emission was 5000 ppbv from the control beer and 8300 ppbv from the “orange peel beer” while a total of 48 and 69 peaks were detected, respectively. Among these peaks, the signals detected at *m*/*z* 79, 109, 123, 125, 133, 141, 149, 151, 153, 157, 161, 163, 165, 167, 169, 171, 177, 183, 185, 187, 203, and 205 were identified only in beers with AEO. All the listed peaks are referred to as terpene compounds or their fragments as reported in Table 2. On the contrary, the control beer showed a small amount of terpenes emissions, which were linked to the addition of small quantities of hops during fermentation.

These compounds, as well as many other VOCs detected (which are common between the two beer samples), showed an increase in emissions due to the addition of AEO. However, other peaks were identified that had an opposite trend and showed a higher emission level in the control beer, such as *m*/*z* 43, 48, 57, 61, 71, 89, and 131. Therefore, we tried to focus our attention on the emission of some alcohols and esters to try to understand how the fermentation behavior of the yeasts throughout the fermentations process was modified by adding AEO. Thus, four flavor-active-compounds were selected, two alcohols (ethanol and isoamyl alcohol) and two esters (ethyl acetate and isoamyl acetate) which directly originate from yeast metabolism during primary fermentation [12].

**Figure 1:**
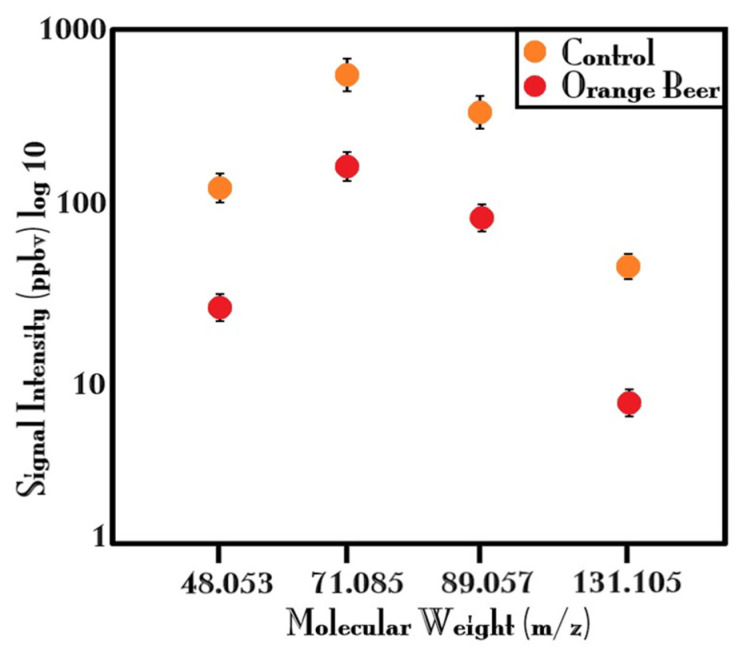
Schematic representation of some aroma compounds directly linked to the yeast fermentation process. The orange point highlights the “Control” sample while the red point highlights the “Orange Beer” sample.

As reported in Figure 1, the ethanol production has been quantified by evaluating its isotope at *m*/*z* 48.053 since the parent ethanol peak at *m*/*z* 47.049 was saturated; the isoamyl alcohol was evaluated following the peak detected at *m*/*z* 71.086; while the esters compounds were assessed at *m*/*z* 89.059 (ethyl acetate) and at *m*/*z* 131.106 (isoamyl acetate), respectively. As shown in Figure 1, adding AEO to beer results in a strong reduction of emissions of compounds linked to the yeast activity.

### 2.3. GC-MS Analysis of Citrus aurantium Essential Oil

**Figure 2:**
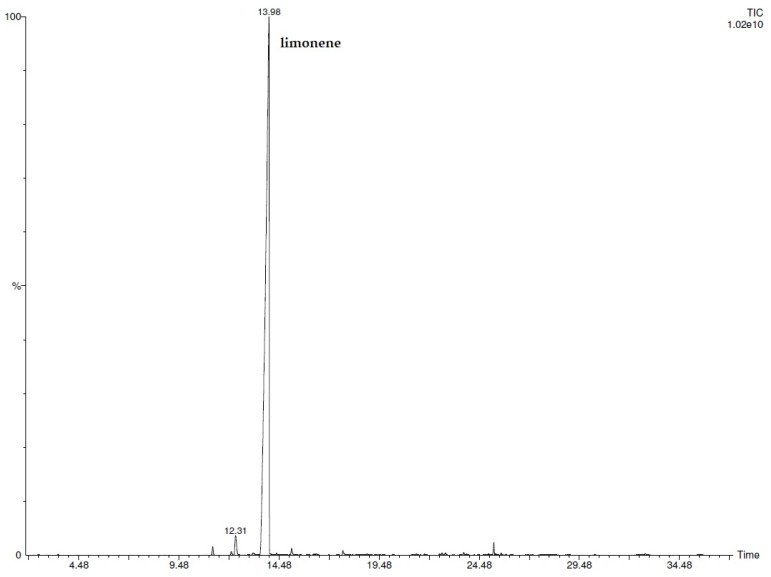
GC-FID chromatogram of AEO.

The chemical characterization of Citrus EO was performed by the GC/MS technique. The monoterpenic component was clearly superior to the sesquiterpenic one due to the absolute prevalence of limonene (95.6%) over the entire volatile fraction. The other terpene compounds detected with a relative percentage greater than 0.1% were *β*-myrcene (1.6%), valencene (0.5%), *α*-pinene (0.4%), linalool (0.4%), and sabinene (0.2%). All the identified components are listed in Table 3. The chromatogram was reported in Figure 2.

### 2.4. Confocal Microscopy Analysis

**Figure 3:**
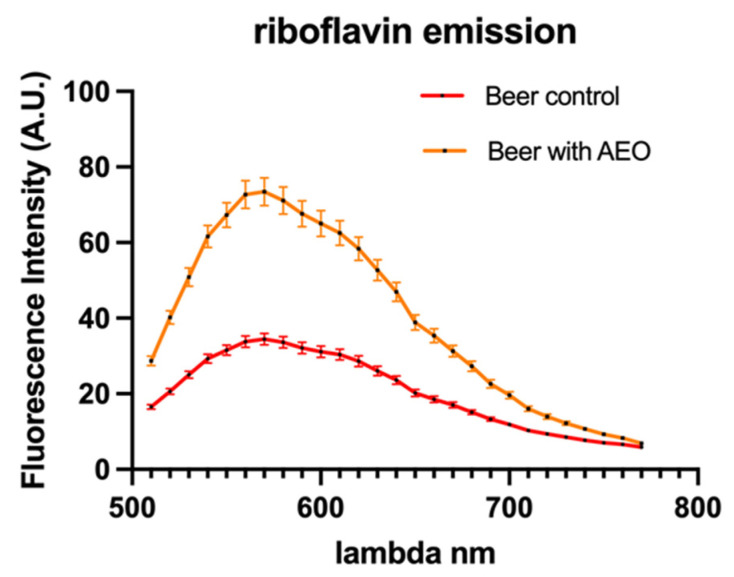
Riboflavin autofluorescence emission spectra detected on yeast cells. (The fluorescence intensity related to riboflavin found in the beer enriched with AEO appeared double compared to the control).

**Figure 4:**
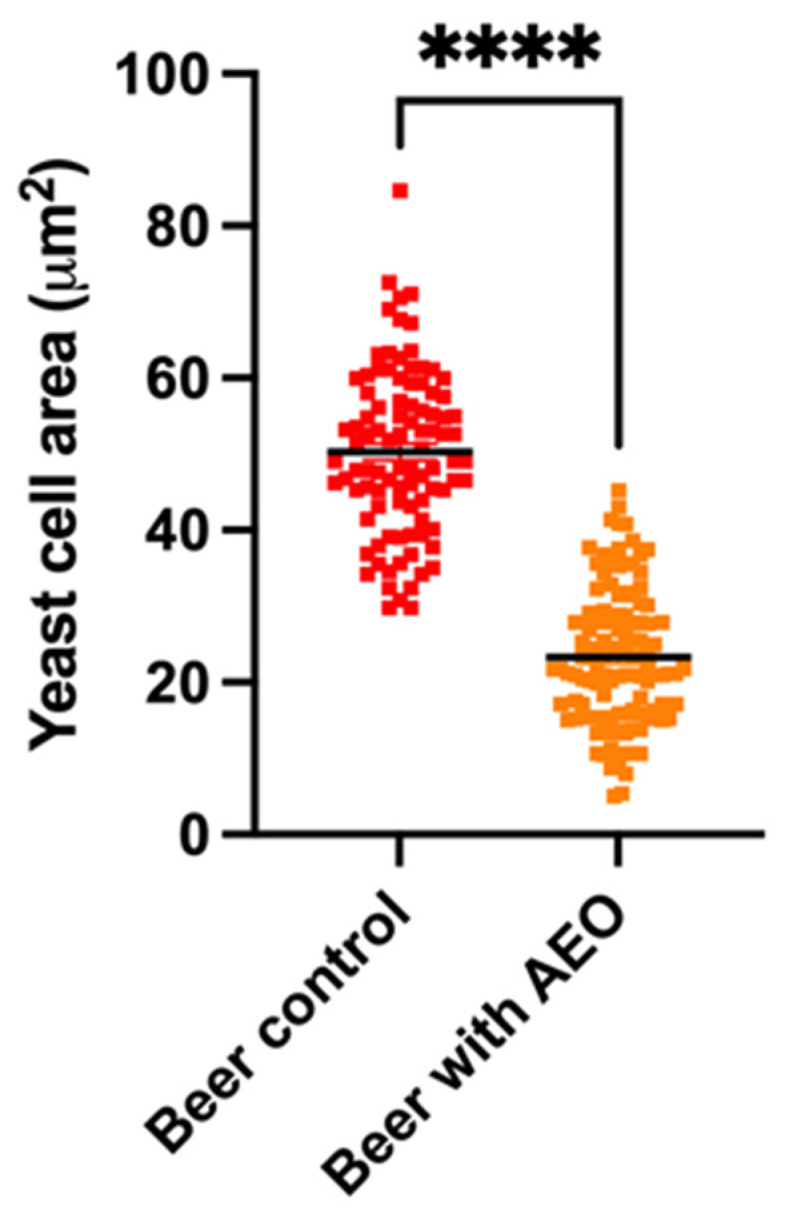
Comparison between yeast cell areas in beer control and those in AEO-enriched beer (lower total cell area than control brewing yeast cells); **** *p* < 0.001.

To examine riboflavin autofluorescence in yeast cells both without and with AEO, we used confocal microscopy. In comparison to the control sample, the amount of fluorescence intensity relative to riboflavin found in the beer enriched with AEO was double (Figure 3). Furthermore, we found that yeast cells co-cultured in beer with AEO had a lower total cell area than control yeast cells (Figure 4).

### 2.5. Spectrophotometric Analysis of Total Polyphenols, Tannins, and Flavonoids

The spectrophotometric analysis showed that the beers had comparable levels of polyphenols and tannins, accounting for roughly 60–70% of the total phenolic compounds; conversely, only traces of flavonoids were detected in both samples (Table 4).

### 2.6. Radical Scavenging Activity

**Figure 5:**
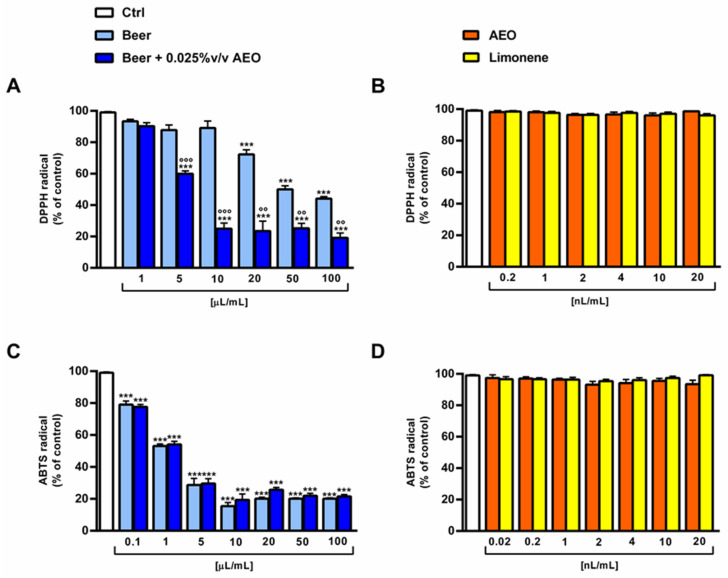
DPPH and ABTS radical scavenger activity of beer and beer + 0.025% *v*/*v* AEO (**A**,**C**) and of AEO and its major compound limonene at the corresponding concentrations in beer (**B**,**D**). Values represent the mean ± SEM (*n* = 6). *** *p* < 0.001 significant difference with respect to Ctrl (one-way ANOVA followed by Dunnett’s multiple comparison post-test). °° *p* < 0.01 and °°° *p* < 0.001 significant difference of beer + 0.025% *v*/*v* AEO with respect to beer (unpaired Student’s *t*-test).

The antioxidant activity of the beers was evaluated in terms of scavenging activity against DPPH and ABTS radicals (Figure 5 and Table 5) in the range of concentrations of 0.01 µL/mL–100 µL/mL. Moreover, AEO and its major compound limonene were tested at corresponding concentrations in the beer (i.e., 0.025% *v*/*v*).

**Figure 6:**
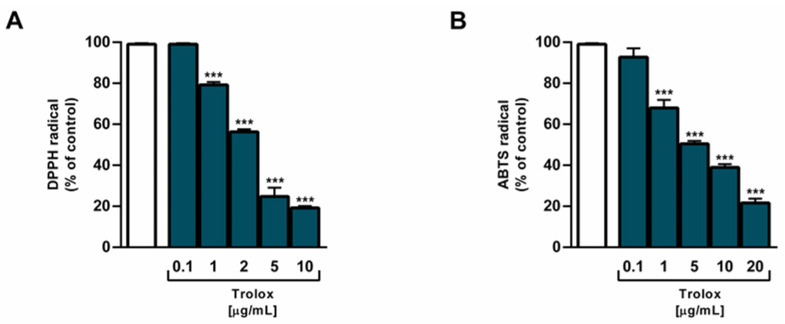
Scavenger activity of the positive control trolox against DPPH (**A**) and ABTS (**B**) radicals. Values represent the mean ± SEM (*n* = 6). *** *p* < 0.001 significant difference with respect to Ctrl (one-way ANOVA followed by Dunnett’s multiple comparison post-test).

Both samples of beer showed radical scavenging properties, with beer + 0.025% *v*/*v* AEO showing a higher potency than beer in the DPPH assay. In fact, despite a 55% DPPH scavenger effect of 10 µL/mL beer, the sample of beer + 0.025% *v*/*v* AEO produced about a 75% DPPH neutralization (Figure 5A). This effect was also confirmed by the IC_50_ value of beer + 0.025% *v*/*v* AEO, which was at least four-folds lower than that of beer (Table 5). By contrast, the samples showed similar scavenger effects towards ABTS radical (Figure 5C), with a significant neutralization of ABTS radical starting from a concentration of 5 µL/mL. Regarding AEO and limonene, they were ineffective towards both radicals at the corresponding concentrations in the beer (i.e., 0.02–20 nL/mL) (Figure 5B,D). As expected, the positive control Trolox displayed a marked and concentration-dependent scavenger activity towards both DPPH and ABTS radicals (Figure 6A,B), and a higher potency than both beer samples (Table 5).

### 2.7. Iron Chelating Activity

**Figure 7:**
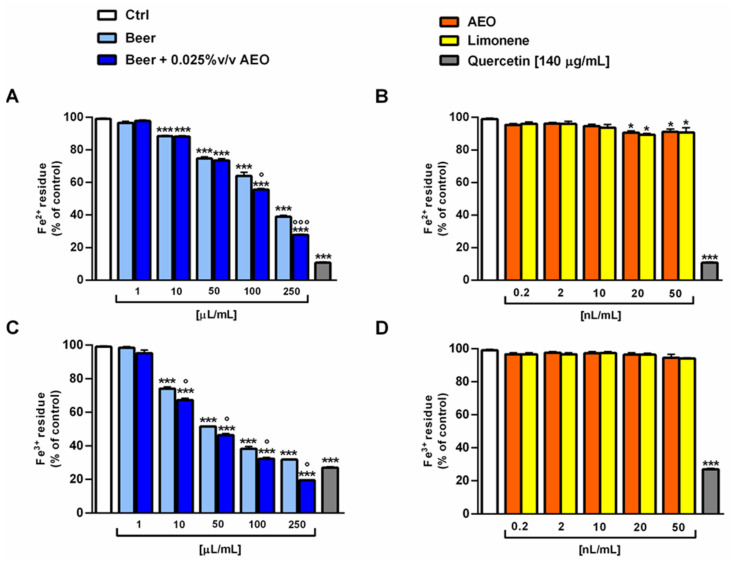
Iron chelating abilities of beer, beer + 0.025% *v*/*v* AEO, and AEO and its major compound limonene at the corresponding concentrations in beer. (**A**) Ferrous ion chelation by the beer samples and the positive control quercetin. (**B**) Ferrous ion chelation by AEO, limonene, and the positive control quercetin. (**C**) Ferric ion chelation by the beer samples and the positive control quercetin. (**D**) Ferric ion chelation by AEO, limonene, and the positive control quercetin. Data represent the average and standard error of at least six replicates obtained in three independent experiments (*n* = 6). * *p* < 0.05 and *** *p* < 0.001 significant difference with respect to Ctrl (one-way ANOVA followed by Dunnett’s multiple comparison post-test). ° *p* < 0.05 and °°° *p* < 0.001 significant difference of beer + 0.025% *v*/*v* AEO with respect to beer (unpaired Student’s *t*-test).

**Figure 8:**
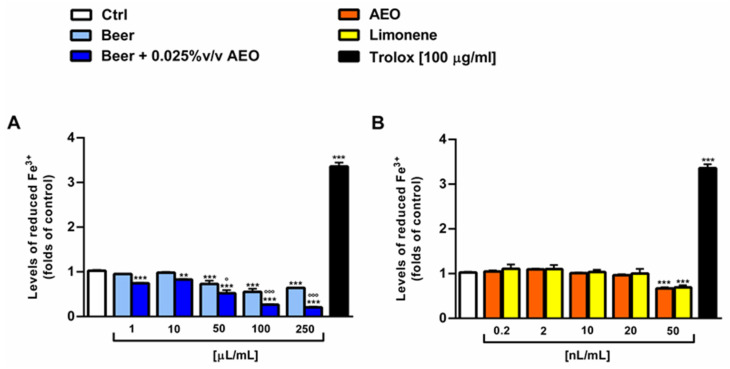
Ferric reducing ability of beer, beer + 0.025% *v*/*v* AEO (**A**), AEO and its major compound limonene (**B**) at the corresponding concentrations in beer, and the positive control trolox. Data represent the average and standard error of at least six replicates obtained in three independent experiments (*n* = 3). *** *p* < 0.001 and ** *p* < 0.001 significant differences with respect to Ctrl (one-way ANOVA followed by Dunnett’s multiple comparison post-test). ° *p* < 0.05 and °°° *p* < 0.001 significant difference of beer + 0.025% *v*/*v* AEO with respect to beer (unpaired Student’s *t*-test).

Under our experimental conditions, the beer samples exhibited similar iron chelating abilities, with a higher potency towards ferric ions (Figure 7A,C). In fact, 50 µL/mL of the beer samples induced a 50% ferric chelation despite a 25% effect on ferrous species. The sample of beer + 0.025% *v*/*v* AEO also exhibited a slight but significant higher chelating power than beer (about 10% increase with respect to beer) starting from the concentrations of 10 and 100 µL/mL towards ferric and ferrous ions, respectively (Figure 7A,C). By contrast, the samples of beer did not show a reduction in ferric ion (Figure 7A). AEO and limonene were ineffective as both iron chelating and reducing agents when assessed at the corresponding concentrations in the beer (i.e., 0.02–50 nL/mL) (Figure 7B,D and Figure 8B). As expected, the known chelating agent quecetin (140 µg/mL) produced almost full iron chelating and reducing activity (Figure 7 and Figure 8).

### 2.8. Antiglycative Activity

**Figure 9:**
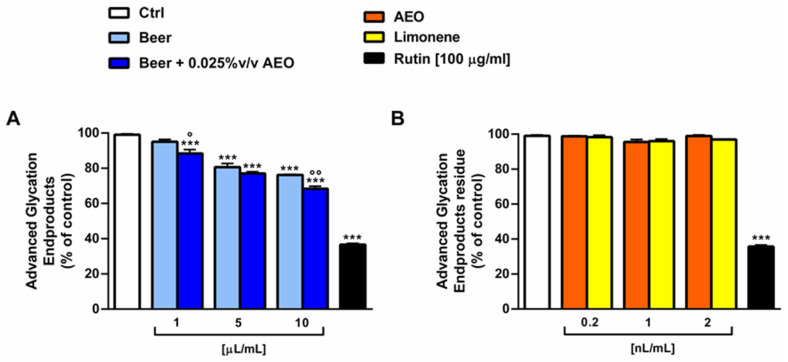
Ability of beer, beer + 0.025 *v*/*v* AEO (**A**), AEO and its major compound limonene (**B**) at the corresponding concentrations in beer, and the positive control rutin to inhibit the formation of advanced glycation end products (AGEs). Values represent the mean ± SEM (*n* = 6). *** *p* < 0.001 significant difference with respect to Ctrl (one-way ANOVA followed by Dunnett’s multiple comparison post-test). ° *p* < 0.05 and °° *p* < 0.01 significant difference of beer + 0.025% *v*/*v* AEO with respect to beer (unpaired Student’s *t*-test).

The antioxidant properties of the samples were also tested in terms of inhibition of the formation of advanced glycation end products (AGEs) that, exerting pro-oxidant oxidative effects, are involved in several pathologies. Under our experimental conditions, both samples of beer showed weak or null AGEs inhibition, achieving a maximum 30% effect at the highest concentration tested, with a slightly higher potency of beer + 0.025% *v*/*v* AEO with respect to beer (Figure 9A). On the other hand, AEO and limonene were ineffective at the corresponding concentrations in the beer (i.e., 0.2–2 nL/mL) (Figure 9B).

### 2.9. Antioxidant Cytoprotective Activity

**Figure 10:**
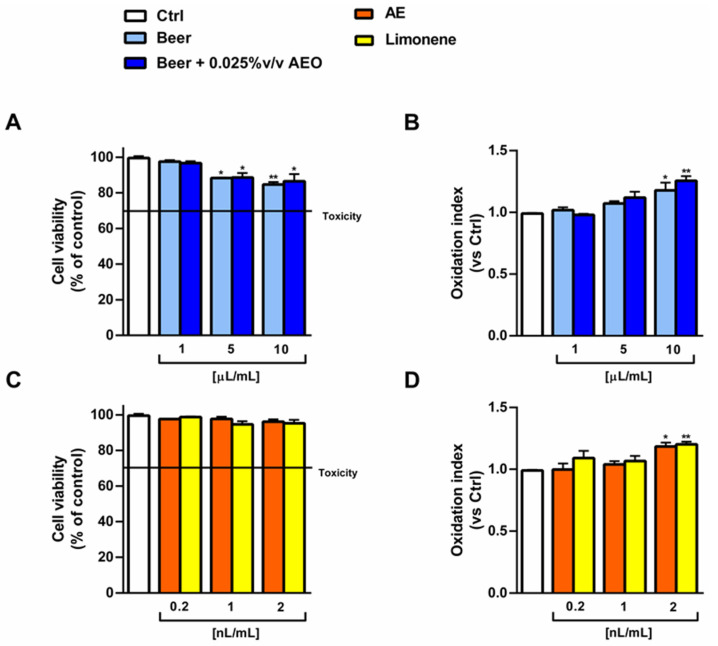
Effect of beer, beer + 0.025% *v*/*v* AEO, AEO and its major compound limonene at the corresponding concentrations in beer on the cell viability (**A**,**C**) and intracellular reactive oxygen species (**B**,**D**) of noncancerous intrahepatic cholangiocytes H69 cells after 24 h exposure. (**A**,**B**) Beer samples. (**C**,**D**) AEO and its major compound limonene. Data are displayed as the mean ± SEM (*n* = 9). (**A**,**C**). Cell viability. (**B**,**D**). Intracellular levels of reactive oxygen species (ROS). * *p* < 0.05 and ** *p* < 0.01 significant difference with respect to Ctrl (one-way ANOVA followed by Dunnett’s multiple comparison post-test).

The beer samples, along with AEO and limonene at the corresponding concentrations in the beer, were also tested for their antioxidant cytoprotective abilities in human noncancerous H69 intrahepatic cholangiocytes towards the pro-oxidant damage induced by tBOOH. Preliminarily, the cytotoxicity of the tested samples was evaluated in order to select the nontoxic concentrations to be studied for cytoprotective activity. Under our experimental conditions, the beer samples did not induce a biologically relevant cytotoxicity up to the highest concentration of 10 µL/mL, thus only weakly affecting cell viability (Figure 10A) and intracellular levels of reactive oxygen species (ROS) (Figure 10B). Similarly, AEO and limonene were nontoxic at the corresponding concentrations in the beer (Figure 10C) but induced a slight oxidative stress with respect to the control, as shown by about a 1.2-fold increase in the ROS levels (Figure 10D). As a result, the cytoprotection towards tBOOH was assessed at the highest concentration of 10 µL/mL.

**Figure 11:**
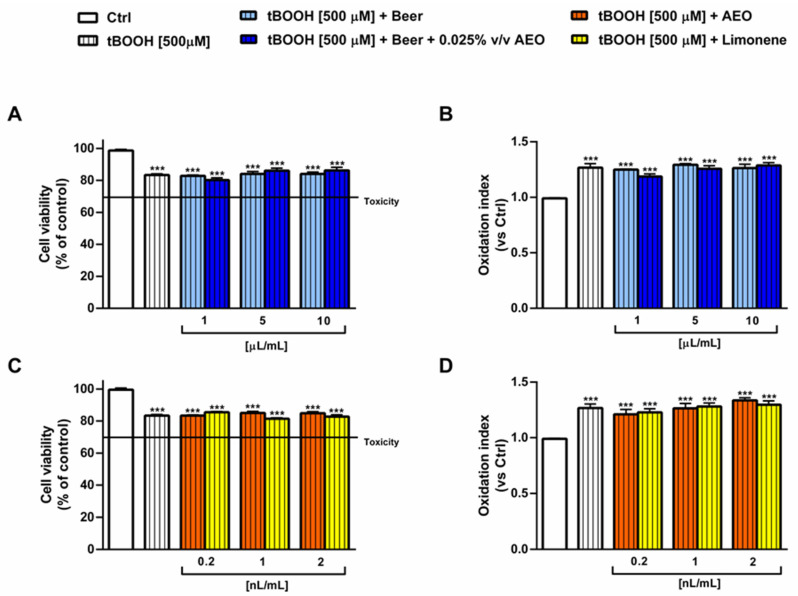
Effect of beer, beer + 0.025% *v*/*v* AEO, AEO and its major compound limonene at the corresponding concentrations in beer on the cell viability (**A**,**C**) and intracellular reactive oxygen species (**B**,**D**) affected by tert-butyl hydroperoxide (tBOOH) in noncancerous intrahepatic cholangiocytes H69 cells. The cells were treated with the samples for 24 h and then with tBOOH for a further 3 h. Data are displayed as the mean ± SEM (*n* = 9). (**A**,**B**) Beer samples. (**C**,**D**) AEO and its major compound limonene. *** *p* < 0.001 significant difference with respect to Ctrl (one-way ANOVA followed by Dunnett’s multiple comparison post-test).

Despite the antioxidant properties highlighted in the radical scavenging and iron chelating assays, the samples of beer did not counteract the oxidative stress induced by tBOOH in H69 cells. In fact, the 20% reduction in cell viability induced by tBOOH was not reversed by the treatments (Figure 11A). As well, the tBOOH-induced ROS intracellular levels were not decreased to the basal levels (Figure 11B). In line with these results, AEO and limonene were ineffective towards tBOOH damage at the corresponding concentrations in beer (Figure 11C,D).

## 3. Discussion

Beer is a very popular low-alcoholic drink which in recent years, given the gradual growing search by consumers for new flavors and aromas, has led producers to create innovative beers deriving from the addition of natural products such as fruit beers [21]. In this context, the aim of this work was to evaluate the effect of adding *Citrus aurantium* essential oil to a gruyt beer on its volatile chemical profile and bioactivity. Both applied analytical techniques showed that the addition of AEO had a significant impact on the beer aroma profile. In fact, compared to the control, beer with AEO was characterized by a higher number of VOCs, a higher total emission of VOCs, and a higher content and emission of terpene compounds. This increase was likely due to the addition of AEO which, as showed by the GC/MS analysis, contained a high number of terpenes with limonene as the predominant compound given the relative percentage above 95%. Terpene compounds are a class of organic compounds responsible for the aroma of many fruits and vegetables. On the other side, in the added beer, the levels of some VOCs which directly originate from yeast metabolism during primary fermentation, such as ethanol, isoamyl alcohol, ethyl acetate, and isoamyl acetate, were lower than in the control.

Confocal microscopy of yeast cells enabled the examination of riboflavin autofluorescence, which in turn elucidated essential information regarding the cofactor riboflavin [22,23]. Several factors, such as pH and the presence of other molecules, can affect riboflavin fluorescence. By using this method, researchers have been able to track riboflavin levels within yeast cells under various experimental conditions [24]. The findings of our investigation suggest that yeast cells grown in the beer gruyt were more likely to be stressed than yeast cells treated with AEO. This is due to the fact that riboflavin is known to play an essential role in the process of ROS detoxification by virtue of its capacity to act as an antioxidant. Hence, one could infer that beer enriched with AEO likely possesses a higher riboflavin content, potentially conferring advantages. At the same time, the smaller size of yeast cells in AEO beer suggests that yeast cell growth is limited compared to control beer. This limitation of yeast cell growth could be advantageous, particularly in cases where the beer is subjected to prolonged bottle conditioning, which could last several months or even years. In such circumstances, curbing yeast cell growth can be beneficial to prevent the beer from excessive carbonation or the development of undesirable off-flavors. Future research could reveal further peculiarities in the interaction between additives and the fermentation process, allowing for the creation of innovative beers characterized by new aromatic profiles.

In the present study, we also evaluated the effect of adding AEO on the antioxidant and cytoprotective properties of beer. *C. aurantium* L. EO has been widely used as a preservative and flavouring agent in the food industry, owing to its organoleptic and antioxidant power [25]. According to our data, limonene has been reported to be the major compound of *C. aurantium* EO from citrus peel, achieving 76% to 95% of the total volatile compounds [25]. This compound is generally recognized as a safe (GRAS) compound and is used as an additive in the food and cosmetic industries too [26].

Several types of beer, both conventional and with the addition of fruits, spices, or natural food during the fermentation process (namely special beer), have been studied for their antioxidant power [27]. These bioactivities may arise from the phytochemicals provided by hops (e.g., bitter acids and polyphenols) during the beer fermentation [28], along with the addition of flavouring and preservative agents to beer for improving its gustatory, olfactory, and visual features [29]. For instance, Pai et al. [30] showed that the total phenolic content of Indian commercial beers, but not flavonoid ones, was positively correlated with the DPPH and ABTS radical scavenging properties and protection in lipid peroxidation. Similar evidence was reported for commercial Portuguese beers [31]. Moreover, Nardini and Foddai [32] found that special beers, obtained by adding natural foods (walnut, chestnut, cocoa, honey, green tea, coffee, and licorice) during the fermentation process, possessed higher ABTS radical scavenging and reducing activities, as well as the content of total polyphenol and flavonoids, than conventional beers [32].

According to this evidence, our results highlighted that beer and beer with AEO were endowed with radical scavenging and iron chelating properties, although with an increased DPPH scavenging power of the AEO beer.

Antioxidant properties were reported for both *C. aurantium* EOs and its major compound limonene. For instance, the EOs from the seven Sicilian cultivars of *C. aurantium* exhibited radical scavenger properties towards DPPH and ABTS radicals (about 30–40 µg/mL IC_50_) higher than those of crops collected in Calabria [25]. Lu et al. [33] also showed that DPPH radical scavenger activity from *Citrus* EOs can be affected by the method of preparation. The radical scavenging properties of limonene are conflicting; in fact, despite the efficacy reported by Shah and Mehta towards DPPH and ABTS radicals (IC_50_ about 385 µM and 603 µM, respectively) [26], Di Sotto et al. [34] did not show radical scavenger properties towards DPPH, ABTS, hydroxyl radical, and superoxide radical, nor chelating properties up to 400 µM.

Under our experimental conditions, despite the bioactivities of the beer samples and the literature, AEO and its major compound limonene were found ineffective at the corresponding concentrations in the beer. Moreover, both beers, AEO and limonene did not show antioxidant properties towards the oxidative damage induced by tBOOH in H69 biliary cells. This evidence suggests that adding 0.025% *v*/*v* AEO to beer may improve its organoleptic features without affecting the potential functional role of this beverage. It could be due to the low amount of AEO and its major compound limonene in the beer with respect to what is needed to provide relevant antioxidant and cytoprotective power.

## 4. Materials and Methods

### 4.1. Plant Material

Organic EO from the peel of *Citrus aurantium* var. *dulcis* L. was provided by the Bergila family business located in Falzes (Bolzano, Italy; 46°48′48.6″ N 11°50′59.6″ E) and stored at 4 °C until use.

### 4.2. Production of Beers

The grist (ground malt grains) recipe (Gruit beer style) was composed of 65% of Pale Ale (Weyermann, Brennerstraße 17-19D-96052 Bamberg, Germany) as base malt, 13.5% Rye malt (Wayermann) for adding fullness or richness to the malt character, 12% hulled oats for softness and foam, 3.5% Cara Pils (Weyermann) for body and foam, and 0.5% toasted chicory for toasted aroma of beer wort.

The mashing process of the ground grains (grains:water in a 1:3 ratio) was performed in a multi-step system. Once the mixture had reached 45 °C, the temperature program proceeded as follows:

(1) 45 °C for 10 min (protease enzymes react to hydrolyze low-weight protein as nourishment for yeast).
(2) 66 °C for 40 min (β-amylase activity, pH 5.0–5.5, enzymatic synergy point between amylases).
(3) 72 °C for 20 min (α-amylase activity, pH 5.6–5.8, maximum activity).
(4) 78 °C for 5 min (enzymatical inactivation phase).

After 15 min of cooling, the filtering took place, with the washing of the threshes and the collection of the wort in a sanitized fermenter. This process was repeated six times with water at pH 6. The wort-boiling phase was performed for seventy minutes.

The hop pellets (Mr. Malt^®^ P.A.B. S.R.L. Via Moretti 4 33037—Pasian di Prato (UD) Italy) used in this recipe were Styrian Golding and were added at the beginning of the boiling phase (28 g in 28 L of must beer).

Subsequently, in the last minute of boiling, taraxacum officinalis (30 g) and nettle (15 g) were used in order to give a characteristic aroma and a more bitter profile. These herbs were supplied by Erbamea srl Via L. Gonzaga 12/A—06016 Selci Lama di San Giustino, PG—Italy.

The beer wort was then cooled during the whirlpooling phase with a counter flow heat exchanger. The cooling phase was performed using a plate-heat exchanger, where the hot mash and the coolant (tap water) circulate in the opposite direction. The mash was then oxygenated to favor the beginning of the fermentation, stirring for at least a couple of minutes.

Finally, the yeast (Fermentis SafAle™ US-05, Lesaffre, Cedex, France) were inoculated and the mix was stirred again. The mix was closed in the fermenter for 12 days at 20 °C, with a gradual temperature decrement down to 4 °C. Lastly, before bottling, 0.25 mL/L of AEO at a temperature of approximately 4 °C was added and appropriately mixed with the beer matrix. Then, the bottling and priming processes were performed. The bottles were stored at 22–25 °C for 20 days. The nucleation of carbon dioxide was then repeated by placing the bottles in a refrigerator at 4 °C for 4–5 days.

### 4.3. Chemicals and Reagents

All the chemicals, including Folin-Ciocalteu’s phenol reagent, polyvinylpyrrolidone (PVP), sodium carbonate, tannic acid (98% purity), aluminum chloride hexahydrate (AlCl_3_·6H_2_O; Ph Eur purity), quercetin (98% purity), 1,1-diphenyl-2-picrylhydrazyl radical (DPPH; 95% purity), 2,2-azobis (2-methylpropionamidine) dihydrochloride (AAPH; 97% purity), 2,2-azino-bis (3-thylbenzothiazoline-6-sulfonic acid) diammonium salt (ABTS; 98% purity), trolox (97% purity), ferrozine (97% purity), iron (II) sulfate heptahydrate (FeSO_4_·7H_2_O; 99% purity), iron (III) chloride (FeCl_3_·6H_2_O; 97% purity), hydroxylamine hydrochloride (98% purity), rutin (99% purity), bovine serum albumin, glucose, fructose, sodium azide, iron (II) chloride (FeCl_2_·4H_2_O; 99% purity), tert-butyl hydroperoxide (tBOOH; 80% purity), and methanol were purchased from Merck (Darmstadt, Germany). RPMI 1640 medium and fetal bovine serum were provided by Aurogene (Rome, Italy).

### 4.4. SPME-GC/MS Analysis of Beers

SPME sampling technique followed by GC/MS analysis was used to describe the volatile profile of the beers. About 2 mL of each beer were placed inside a 15 mL glass vial with a PTFE-coated silicone septum. A SPME device from Supelco (Bellefonte, PA, USA) with 1 cm fiber coated with 50/30 μm DVB/CAR/PDMS (divinylbenzene/carboxen/polydimethylsiloxane) was chosen to extract the components. The operative conditions followed Taiti et al. [23]. Component desorption was ensured by inserting the fiber directly into the GC injector maintained at 250 °C in splitless mode.

The chromatographic analyses of the headspace beers were carried out by Clarus 500 model Perkin Elmer (Waltham, MA, USA) gas chromatograph coupled with a mass spectrometer equipped with a FID (flame detector ionization) and with a Varian (VF-1ms; 60 m × 0.32 mm × 1 μm, Agilent, Santa Clara CA, USA) apolar column. The operative conditions were as reported in previous works [23,35]. Briefly, the oven programmed temperature was set initially at 55 °C and then increased to 220 °C at 6°/min and finally held for 15 min. Helium was used as carrier gas at a constant rate of 1 mL/min. MS detection was performed with electron ionization (EI) at 70 eV operating in the full-scan acquisition mode in the *m*/*z* range 30–450 amu. To identify the volatile compounds, the MS-fragmentation pattern obtained was compared with those of pure components stored in the Nist 11 mass spectra library database. The Linear Retention Indices (LRIs) were also calculated using a series of alkane standards (C_8_–C_25_ *n*-alkanes) and then compared with those available in the literature. Relative percentages for quantification of the components were calculated by electronic integration of the GC-FID peak areas and no response factors were calculated. Analyses were carried out in triplicate.

### 4.5. GC/MS Analysis of AEO

To characterize the chemical composition of AEO, the same apparatus as described above and the same column were used. 1 µL of the EO was diluted in 1 mL of methanol and 1 µL of the solution was injected into the injector at 270 °C with a 1:20 split. Helium was used as carrier gas at a constant rate of 1 mL/min. The GC oven temperature program was followed as described by Ovidi et al. [36], with a slight modification whereby from 60 °C it was ramped to 220 °C at a rate of 6°/min and finally isothermal at 220 °C for 25 min. The Electron Impact-Mass Spectrometer (EI-MS) mass spectra were recorded at 70 eV (EI) and scanned in the range 40–550 *m*/*z*. The identification and quantification of the components were performed as reported in the previous Section 4.4.

### 4.6. PTR-ToF-MS Analysis of Beers

Headspace measurements of beer aroma were performed with the PTR-MS 8000 model (IONICON Analytik, Innsbruck, Austria), which was operated in its standard operational setting (V mode) using H_3_O^+^ as an H^+^ donor. Tool configuration and analysis methods were conducted following the setting previously described by Taiti and co-authors in a previous work on beer samples [23]. Since the high ethanol concentration in beer could affect the final quantification of volatiles, argon was directly injected into the drift tube with the aim of diluting the samples (1:2) as suggested by Campbell-Sills and co-authors [37]. Therefore, all the peaks linked to ethanol and ethanol clusters (*m*/*z* 29, 30, 32, 34, 37, 39, 46, 47, 55, 65, 66, 75, 76, 93, 94, 121, 122, 139) were discarded excluding *m*/*z* 28 and 48 [37]. For each sample, the headspace analysis took place for 100 consecutive seconds, with an acquisition time equal to 1 spectrum per second between a range of *m*/*z* 15 and 220. Data were acquired throughout TOFDAQ v.183 and were expressed as ppb/v (parts per billion per volume). After each run, the internal calibration was performed off-line following the procedure previously described by Cappellin et al. [38]. Thus, a noise reduction of the dataset was conducted by eliminating all signals whose average concentration of three samples was lower than 1 ppb/v. Thereafter, the tentative identification of peaks detected was based on the following: (a) previous PTR studies on beer or other alcoholic products; (b) the main terpenoids pattern fragmentation available in the PTR literature as proposed by some authors (Table 2).

### 4.7. Confocal Microscopy of Beer Yeast Cells

Fluorescence spectra were obtained from autofluorescence relative to riboflavin using a confocal microscope [22,23]. Leica TCS SP5 confocal microscope (Leica Microsystems CMS, Wetzlar, Germany) fitted with an acousto-optical beam splitter and an upright microscope stand was used to capture the spectral images (DMI6000). Images of a drop of beer and beer infused with orange peel were captured using a 40× objective (HCX PL APO OIL UV) and a 98 mm × 98 mm frame size. The pinhole was set to one ‘Airy unit’. Fluorescence spectra of riboflavin were recorded (using the Leica LAS-AF software package, over the 510–770 nm waveband) by performing measurements in the λ-scan mode with a detection window of 10 nm and a 488 nm laser as the excitation line. This allowed for the collection of data over the entire waveband. The program Fiji (https://imagej.net/Fiji, accessed on 30 September 2023) was used to measure the surface area of yeast cells [39].

### 4.8. Determination of Total Polyphenols, Tannins, and Flavonoids

The content of total polyphenols and tannins in the beer samples was assessed through the spectrophotometric method of Folin-Ciocalteu, according to previously published methods [40]. The amount of total phenolics was measured at 765 nm and expressed as mg of tannic acid equivalents (TAE) per mL of beer. The content of flavonoids was evaluated through aluminum chloride and expressed as mg of quercetin equivalents (QEs) per mL of beer [40]. The equations of calibration curves for tannic acid and quercetin, calculated by linear regression (GraphPad Prism™ 8.0.1), were Y = 14314X + 0.05686 (r^2^ = 0.975) and Y = 17903X − 0.001203 (r^2^ = 0.995), respectively.

### 4.9. Radical Scavenger Activities

DPPH- and ABTS-radical scavenging activities of the tested samples, i.e., beer, beer with AEO 0.025% *v*/*v*, AEO at the corresponding volumes in the beer, and the major compound in AEO, were assessed according to previously described spectrophotometric methods [34]. The assays were performed by measuring the residue of DPPH and ABTS radicals at 517 nm and 734 nm, respectively. As a positive control, progressive concentrations of trolox were used. The percentage of scavenger activity was calculated as follows: 100 × (Acontrol − Asample)/Acontrol, where Acontrol is the absorbance of the radical alone, while Asample is that of radical with sample.

### 4.10. Chelating Activity

Chelating activity of the tested samples towards both ferrous and ferric ions was evaluated by the ferrozine assay, according to previously published spectrophotometric methods and using quercetin and trolox as positive controls, respectively [41]. The percentage of activity was calculated as follows: 100 × (Acontrol − Asample)/Acontrol, where Acontrol is the absorbance of the vehicle while Asample is that of the tested sample.

### 4.11. Ferric Ion Reducing Activity

The ferric ion reducing activity of the tested samples was evaluated by the ferrozine assay, using trolox as a positive control [41]. The levels of reduced iron were calculated as a percentage of the vehicle control as follows: 100 × (Asample)/Acontrol, where Asample is that of the tested sample while Acontrol is the absorbance of the vehicle.

### 4.12. Inhibition of Advanced Glycation End-Product (AGE)

The ability to counteract the formation of AGE was assessed by performing a previously published fluorescent method [42]. Fluorescence was measured at an excitation wavelength of 355 nm and an emission of 460 nm. The flavonoid rutin was used as standard antiglycative agent. The activity was evaluated as a percentage of the control by using the following formula: (Acontrol − Asample/Acontrol) × 100, where Acontrol is the fluorescence of the control, whereas Asample is the fluorescence of the sample.

### 4.13. Cytoprotective Activity under Oxidative Stress

#### 4.13.1. Cell Culture

Noncancerous intrahepatic H69 cholangiocytes was used as an in vitro model of the gastro-intestinal tract to study the possible cytoprotective properties of the samples. H69 cells were kindly provided by Romina Mancinelli (Department of Anatomical, Histological, Forensic and Orthopedic Sciences, Sapienza University of Rome, Italy) and were grown under standard conditions (37 °C and 5% CO_2_), according to previously published methods [23]. The culture media quality was periodically monitored for purity and absence of possible microbiological contaminations, according to previously published methods [43,44]. All experiments were performed when the cells reached about an 80% confluence.

#### 4.13.2. Cytotoxicity Assay

Confluent cells were seeded into 96-well microplates (2 × 10^4^ cells/well) and then exposed for 24 h to progressive volumes of the tested samples, i.e., beer, beer with AEO 0.025% *v*/*v*, AEO at the corresponding volumes in the beer, and the major compound in AEO, up to a maximum 1% *v*/*v* in the medium. Considering that the tested beers were 5% *v*/*v* alcohol grade, a maximum 0.05% (*v*/*v*) EtOH in cell medium was used as a vehicle control. Accordingly, the essential oil and its major compound limonene, along with the positive control doxorubicin were assayed. Cell viability was evaluated by the 3-[4,5-dimethylthiazol-2-yl]-2,5-diphenyl tetrazolium bromide (MTT) assay, by detecting the formazan absorbance at 595 nm, according to previously published methods [23]. For each treatment, the cell viability was expressed as percentage of viable cells with respect to the vehicle control (treated with 0.05% (*v*/*v*) EtOH). A higher than 30% reduction in cell viability with respect to the control was considered as a biologically significant cytotoxic effect of the treatments [45].

#### 4.13.3. Cytoprotection towards the Oxidative Damage Induced by Tert-Butyl Hydroperoxide (tBOOH)

The cytoprotective abilities of the tested samples were assessed against the oxidative damage induced by tBOOH (500 μM) [23]. To this end, a subtoxic concentration, which induced about a 40% inhibition of the cell viability in previous experiments, was used. Briefly, 2 × 10^4^ cells/well were grown in a 96-well microplate for 24 h and then exposed to the tested samples for 24 h and to the tested samples and tBOOH for a further 3 h. Changes in cell viability and intracellular levels of reactive oxygen species (ROS) were measure by the MTT and 2,7-dichlorofluorescein diacetate (DCFH-DA) assays, respectively [42].

### 4.14. Statistical Analysis

Data represent the average and standard error (SE) of at least six replicates obtained in three independent experiments (*n* = 6). GraphPad Prism™ (Version 6.00) software (GraphPad Software, Inc., San Diego, CA, USA) to perform the statistical analysis and data representation. Preliminarily, data were assessed for normality distribution by the D’Agostino and Pearson omnibus normality test (GraphPad Prism™ 6.00). A statistically significant difference (*p* value < 0.05) of the treatments with respect to the vehicle control was evaluated by the one-way analysis of variance (one-way ANOVA), followed by Dunnett’s Multiple Comparison Post Test, while that of beer + 0.025% *v*/*v* AEO with respect to beer by the unpaired Student’s *t*-test.

Values obtained by confocal microscopy are presented as the mean SEM of the number of the indicated determinations. Statistical significance was determined using an unpaired *t*-test (PRISM 9.0; GraphPad Software), with *p* ≤ 0.005 considered significant.

## 5. Conclusions

In this work, the changes induced by the addition of essential oil of *Citrus aurantium* to Gruyt craft beer were evaluated both in terms of composition and biological activity. The results obtained from the chemical analyses demonstrated that the beer with AEO had a wider range of VOCs which conferred a characteristic profile thanks also to the predominant presence of limonene. Confocal microscopy highlighted that the yeast cells were smaller in the added beer but also likely showed greater resistance to oxidative stress due to the high amount of riboflavin detected. Furthermore, the results obtained from the biological assays confirmed that the addition of AEO to beer induced a moderate increase in its DPPH removal and chelation capabilities without affecting the cytoprotective properties.

Further studies would also be useful to better understand the appropriate allowed quantities of added bioactive compounds to confer peculiar properties to beer without altering its fundamental characteristics.

## Figures and Tables

**Table 1:** Headspace chemical volatile composition (percentage mean values ± standard deviation) of beers by HS-SPME-GC/MS.

N°	Component ^1^	LRI ^2^	LRI ^3^	Beer (%)	Beer + AEO(%)
**1**	1-propanal, 2-methyl-	528	537	9.0 ± 0.0	tr
**2**	isoamyl alcohol	738	740	10.1 ± 0.1	tr
**3**	active amyl alcohol	741	744	17.0 ± 0.1	tr
**4**	isoamyl acetate	860	858	6.7 ± 0.0	-
**5**	*α*-pinene	928	932	-	0.3 ± 0.0
**6**	*β*-myrcene	982	987	-	1.3 ± 0.0
**7**	*α*-phellandrene	1002	1000	-	tr
**8**	limonene	10,032	1029	2.0 ± 0.0	96.6 ± 14.1
**9**	*γ*-terpinene	1046	1048	-	0.1 ± 0.0
**10**	terpinolene	1081	1084	-	0.2 ± 0.0
**11**	ethyl caprylate	1178	1181	47.6 ± 1.1	0.4 ± 0.0
**12**	*β*-elemene	1377	1381	-	tr
**13**	ethyl caprate	1381	1382	5.6 ± 0.0	0.1 ± 0.0
**14**	butyl caprylate	1390	1388	2.0 ± 0.0	-
**15**	*β*-caryophyllene	1444	1440	-	0.2 ± 0.0
**16**	gemacrene D	1491	1489	-	tr
**17**	humulene	1476	1473	-	tr
**18**	*β*-eudesmene	1485	1481	-	0.4 ± 0.0
**20**	valencene	1516	1515	-	tr
**19**	*δ*-cadinene	1519	1520	-	tr
**21**	*α*-panasinsen	1531	1527	-	0.1 ± 0.0
	**SUM**			100.0	99.7

^1^ The components are reported according to their elution order on apolar column; ^2^ Linear Retention Indices measured on apolar column; ^3^ Linear Retention indices from the literature; - Not detected; tr: percentage mean values <0.1%.

**Table 2:** Volatile organic compounds detected by PTR-ToF-MS analysis. Data are reported as the average of three replicates ± DS and are expressed as ppb/v: compounds number, mass/charge (*m*/*z*) ratios, chemical formula, and tentative identifications for each VOCs detected. A tentative identification was made by comparing each peak with those previously reported in the beer literature or by the terpenoids pattern provide by the PTR literature. At the bottom were also reported a total VOCs emission, a total terpenoid emission, and a number of signals detected for each sample type.

Compounds Number	*m*/*z*	Chemical Formulae (H^+^)	Tentative Identification (TI)	Reference	Control Beer (±SD)	Beer + AEO (±SD)
**1**	28.006	C_2_H_5_^+^	Ethanol Fragment	[12]	208.0 ± 82.3	117.7 ± 36.6
**2**	41.038	C_2_H_5_^+^	Fragment (alcohol, ester)		272.3 ± 68.6	338.8 ± 27.8
**3**	43.018	C_2_H_3_O^+^	Propene (fragment)	[13]	513.2 ± 72.3	298.2 ± 42.2
**4**	43.054	C_2_H_7_^+^	Hydrocarbon fragments	[13]	614.2 ± 20.1	348.2 ± 39.3
**5**	48.053	C_2_H_8_O	Ethanol isotope	[12]	162.6 ± 19.1	45.1 ± 12.7
**6**	51.028	C_4_H_3_^+^	C4 fragment		10.8 ± 3.8	6.3 ± 2.5
**7**	53.038	C_4_H_5_^+^	Alkyl fragment	[14]	2.3 ± 0.2	113.6 ± 16.1
**8**	57.069	C_4_H_9_^+^	Hydrocarbon fragments	[13]	511 ± 44.4	129.6 ± 21.2
**9**	59.049	C_3_H_7_O^+^	Acetone/propanal		215.2 ± 16.7	773.1 ± 69.4
**10**	61.029	C_2_H_5_O_2_^+^	Acetic acid		440 ± 52.1	176.6 ± 29.2
**11**	63.027	C_2_H_7_S^+^	S compounds (dimethylsulfide)		44.8 ± 19.1	29.2 ± 11.1
**12**	65.059	C_2_H_9_O_2_^+^	Alkyl fragment		21.2 ± 7.2	12.3 ± 5.7
**13**	67.054	C_5_H_7_^+^	Terpene fragment	[15]	2.6 ± 0.3	658.4 ± 49.5
**14**	69.069	C_5_H_9_^+^	Terpene fragment	[16]	12.6 ± 1.3	368.5 ± 27.7
**15**	71.085	C_5_H_11_^+^	Isoamyl alcohol	[12]	588.6 ± 61.3	214.5 ± 29.1
**16**	73.064	C_4_H_9_O^+^	Butanal		24.8 ± 2.6	24.7 ± 1.9
**17**	79.054	C_6_H_7_^+^			-	471.7 ± 35.4
**18**	81.069	C_6_H_9_^+^	Terpene fragment	[16]	8.8 ± 1.2	757.3 ± 56.9
**19**	83.049	C_5_H_7_O^+^			9.7 ± 1.4	13.2 ± 1.0
**20**	85.064	C_5_H_9_O^+^	Pentanal/pentenone	[12]	16.4 ± 2.3	16.1 ± 2.6
**21**	87.080	C_5_H_11_O^+^	Pentanol	[12]	24.1 ± 9.4	14.1 ± 5.1
**22**	89.057	C_5_H_13_O^+^	Ethyl acetate	[12]	396.2 ± 67.0	123.6 ± 18.5
**23**	91.057	C_7_H_7_^+^	Terpene fragment	[15]	8.8 ± 1.2	34.3 ± 2.6
**24**	95.096	C_7_H_11_^+^	Terpene fragment	[16]	10.1 ± 1.4	379.7 ± 28.5
**25**	99.080	C_6_H_11_O^+^	Hexanol	[12]	5.4 ± 0.3	6.6 ± 0.9
**26**	101.059	C_5_H_9_O_2_^+^			11.7 ± 1.2	12.9 ± 1.8
**27**	103.073	C_5_H_11_O_2_^+^	Ethyl propionate, or methyl butanoate	[13]	40.7 ± 8.6	32.1 ± 10.7
**28**	105.070	C_8_H_9_^+^	Phenethyl alcohol	[12]	118.5 ± 18.2	81.3 ± 11.2
**29**	107.086	C_8_H_11_^+^	Terpene fragment	[17]	18.7 ± 2.6	221.0 ± 31.3
**30**	109.101	C_8_H_13_^+^	Terpene fragment	[16]	-	179.8 ± 25.9
**31**	111.080	C_7_H_11_O^+^	Heptadienal	[12]	5.8 ± 1.4	176.9 ± 24.9
**32**	113.096	C_6_H_13_O^+^	Heptanal	[12]	5.3 ± 0.8	46.1 ± 6.5
**33**	115.075	C_7_H_15_O^+^			9.0 ± 1.3	33.2 ± 4.7
**34**	117.090	C_6_H_13_O_2_^+^	Hexanoic acid, or methyl pentanoate	[13]	121. ± 1.0	79.7 ± 11.0
**35**	119.086	C_9_H_11_^+^	Terpene fragment	[17]	1.4 ± 0.2	59.3 ± 8.4
**36**	123.118	C_9_H_15_^+^	Terpene fragment	[16]	-	113.9 ± 16.1
**37**	125.096	C_8_H_13_O^+^			-	29.6 ± 4.2
**38**	127.075	C_7_H_11_O_2_^+^			1.1 ± 0.2	170.8 ± 24.2
**39**	129.091	C_7_H_13_O_2_^+^	Esters and acids	[12]	9.3 ± 2.8	17.1 ± 6.4
**40**	131.105	C_7_H_15_O_2_^+^	Isoamyl acetate	[12]	44.3 ± 6.3	11.2 ± 2.2
**41**	133.101	C_10_H_13_^+^	p-cymenene		-	29.5 ± 4.2
**42**	135.116	C_10_H_15_^+^	Terpenoids (e.g., p-cymene)	[16]	4.9 ± 0.7	33.3 ± 4.7
**43**	137.132	C_10_H_17_^+^	Terpenoids (e.g., limonene)	[16]	1.8 ± 0.2	122.1 ± 17.3
**44**	141.163	C_10_H_21_^+^	Terpenes fragments	[14]	-	2.7 ± 0.4
**45**	143.146	C_9_H_19_O^+^	Nonanone/nonanal	[12]	9.7 ± 2.2	24.6 ± 3.5
**46**	145.121	C_8_H_17_O_2_^+^	Ethyl hexanoate	[12]	61.6 ± 14.1	36.6 ± 9.2
**47**	149.132	C_11_H_17_^+^	Terpenoids	[18]	-	66.5 ± 9.4
**48**	151.112	C_10_H_15_O^+^	Terpenoids (e.g., D-Verbenone)	[18]	-	173.4 ± 24.5
**49**	153.153	C_10_H_17_O^+^	Terpenoids (e.g., citral)	[15]	-	419.9 ± 49.4
**50**	157.159	C_10_H_21_O^+^	Terpenoids (e.g., citronellol)	[14]	-	6.8 ± 1.0
**51**	159.137	C_9_H_19_O_2_^+^	C9 ester/nonanoic acid	[19]	2.2 ± 0.2	2.6 ± 0.4
**52**	161.153	C_9_H_21_O_2_^+^	Terpene oxidation products	[20]	-	25.4 ± 3.6
**53**	163.148	C_12_H_19_^+^	Terpenoids (e.g., aromandrene)	[16]	-	22.5 ± 3.2
**54**	165.125	C_11_H_17_O^+^	Terpenoids (e.g., Santalone)	[20]	-	38.3 ± 5.4
**55**	167.106	C_10_H_15_O_2_^+^	Terpene oxidation products	[20]	-	41.1 ± 3.1
**56**	169.122	C_10_H_17_O_2_^+^	Terpenoids (e.g., iridomyrmecin)		-	21.2 ± 1.6
**57**	171.137	C_10_H_19_O_2_^+^	Terpenoids (e.g., hydroxygeraniol)	[15]	-	21.0 ± 1.6
**58**	173.153	C_10_H_21_O_2_^+^	Ethyl caprylate/Ethyl octanoate	[12]	134.7 ± 16.5	131.4 ± 9.1
**59**	175.170	C_10_H_23_O_2_^+^			32.4 ± 0.3	38.5 ± 3.6
**60**	177.165	C_13_H_21_^+^			-	11.7 ± 0.9
**61**	185.132	C_14_H_17_^+^	Terpenoids (e.g., chamazulene)		-	27.2 ± 2.0
**62**	187.170	C_11_H_23_O_2_^+^			3.3 ± 1.4	5.8 ± 0.4
**63**	189.185	C_11_H_25_O_2_^+^			1.8 ± 0.5	9.9 ± 0.7
**64**	201.190	C_12_H_25_O_2_^+^	Ethyl caprate/Butyl caprylate		3.1 ± 0.3	13.5 ± 1.0
**65**	203.179	C_15_H_22_^+^	Terpenoids (e.g., eudesmatriene)	[20]	-	24.7 ± 1.8
**66**	205.195	C_15_H_24_^+^	Terpenoids (e.g., cedrene)	[16]	-	67.3 ± 5.0
**67**	28.006	C_2_H_5_^+^	Ethanol Fragment	[12]	208.0 ± 82.3	117.7 ± 36.6
**52**	41.038	C_2_H_5_^+^	Fragment (alcohol, ester)		272.3 ± 68.6	338.8 ± 27.8
**53**	43.018	C_2_H_3_O^+^	Propene (fragment)	[13]	513.2 ± 72.3	298.2 ± 42.2
**Total VOCs emission (average ± DS)**					4780.2 ± 686.8	8169.4 ± 940.3
**Total Terpenoids emission (average ± DS)**					69.7 ± 9.3	3915.0 ± 384.5
**Total signals detected (>1pbbv)**					46	67

**Table 3:** Chemical volatile composition (percentage mean value ± standard deviation) of essential oil by GC-MS.

N°	Component ^1^	LRI ^2^	LRI ^3^	(%)
**1**	*α*-pinene	928	932	0.4 ± 0.0
**2**	sabinene	968	972	0.2 ± 0.0
**3**	*β*-myrcene	982	987	1.6 ± 0.0
**4**	limonene	1032	1029	95.3 ± 12.1
**5**	*γ*-terpinene	1045	1048	0.1 ± 0.0
**6**	linalool	1089	1095	0.4 ± 0.0
**7**	*p*-mentha-*trans*-2,8-dien-1-ol	1100	1103	tr
**8**	limonene epoxide	1134	1138	tr
**9**	citronellal	1140	1146	0.1 ± 0.0
**10**	decanal	1183	1186	0.3 ± 0.0
**11**	carvone	1222	1226	0.1 ± 0.0
**12**	*α*-citral	1281	1287	tr
**13**	undecanal	1305	1309	tr
**14**	nerol acetate	1344	1342	0.1 ± 0.0
**15**	*α*-copaene	1387	1385	0.1 ± 0.0
**16**	dodecanal	1392	1399	tr
**17**	*β*-caryophyllene	1438	1440	0.1 ± 0.0
**18**	isogermacrene D	1447	1446	0.1 ± 0.0
**19**	humulene	1468	1473	tr
**20**	germacrene D	1486	1489	tr
**21**	*α*-selinene	1510	1512	tr
**22**	valencene	1516	1515	0.5 ± 0.0
**23**	*δ*-cadinene	1519	1520	0.1 ± 0.0
**24**	*α*-panasinsen	1522	1527	tr
**25**	elemol	1537	1535	tr
	**SUM**			99.6

^1^ the components are reported according to their elution order on apolar column; ^2^ Linear Retention Indices measured on apolar column; ^3^ Linear Retention indices from the literature.

**Table 4:** Total amount of polyphenols, tannins, and flavonoids in beer and beer + 0.025 *v*/*v* AEO. Data are expressed as means ± standard error (SE) of at least two experiments and six replicates.

Sample	Total Polyphenols	Tannins	Flavonoids
	mg TAE/mL Beer		mg QE/mL Beer
Beer	2.1 ± 0.1	1.5 ± 0.1	0.04 ± 0.02
Beer + 0.025% *v*/*v* AEO	2.2 ± 0.2	1.3 ± 0.2	0.03 ± 0.00

TAE: tannic acid equivalents; QE: quercetin equivalents.

**Table 5:** IC_50_ values (µL/mL) of beer, beer + 0.025% *v*/*v* AEO (Figure 5A,C), AEO and its major compound limonene at the corresponding concentrations in beer, and the positive control trolox (µg/mL) in DPPH and ABTS radical scavenging activity assays.

Sample		DPPH Scavenging Activity	ABTS Scavenging Activity
Beer	IC_50_ (CL) µL/mL	23.9 (16.3–35.1)	12.1 (7.1–20.6)
Beer + 0.025% *v*/*v* AEO		5.2 (4.3–6.3) ^#^	11.6 (8.3–16.2)
AEO		ne	ne
Limonene		ne	ne
Trolox	IC_50_ (CL) µg/mL	5.1 (0.5–26.7)	4.1 (1.0–15.8)

CL: confidence limit; ne: not evaluable as the achieved effect at the tested concentration was lower than 80%; ^#^ *p* < 0.05, significant difference of beer + 0.025% *v*/*v* AEO with respect to beer (unpaired Student’s *t*-test).

## Data Availability

All generated data are included in this article.

## References

[1] Palamand S.R., Aldenhoff J.M. (1973). Bitter tasting compounds of beer: Chemistry and taste properties of some hop resin compounds. J. Agric. Food Chem..

[2] Villacreces S., Blanco C.A., Caballero I. (2022). Developments and characteristics of craft beer production processes. Food Biosci..

[3] Cimino C., Maurel O.M., Musumeci T., Bonaccorso A., Drago F., Barbosa Souto E.M., Pignatello R., Carbone C. (2021). Essential Oils: Pharmaceutical Applications and Encapsulation Strategies into Lipid-Based Delivery Systems. Pharmaceutics.

[4] Sharmeen J.B., Mahomoodally F.M., Zengin G., Maggi F. (2021). Essential Oils as Natural Sources of Fragrance Compounds for Cosmetics and Cosmeceuticals. Molecules.

[5] Saeed K., Pasha I., Farhan M., Chughtai J., Ali Z., Bukhari H., Zuhair M. (2022). Application of essential oils in food industry: Challenges and innovation. J. Essent. Oil Res..

[6] Mith H., Dure R., Delcenserie V., Zhiri A., Daube A., Clinquart A. (2014). Antimicrobial activities of commercial essential oils and their components against food-borne pathogens and food spoilage bacteria. Food Sci. Nutr..

[7] Arcoleo G., Indovina M.C., Varvara G., Lanza C.M., Mazzaglia A. (2009). Improving olive oil shelf life with lemon essential oil. Chem. Eng. Trans..

[8] Caputi L., Aprea E. (2011). Use of Terpenoids as Natural Flavouring Compounds in Food Industry. Recent Pat. Food Nutr. Agric..

[9] Chanthaphon S., Chanthachum S., Hongpattarakere T. (2008). Antimicrobial activities of essential oils and crude extracts from tropical *Citrus* spp. against food-related microorganisms. Songklanakarin J. Sci. Technol..

[10] Fisher K., Phillips C. (2008). Potential antimicrobial uses of essential oils in food: Is citrus the answer?. Trends Food Sci. Technol..

[11] Calo J.R., Crandall P.G., O’Bryan C.A., Ricke S.C. (2015). Essential oils as antimicrobials in food systems—A review. Food Control.

[12] Roberts R., Khomenko I., Eyres G.T., Bremer P., Silcock P., Betta E., Biasioli F. (2023). Online monitoring of higher alcohols and esters throughout beer fermentation by commercial Saccharomyces cerevisiae and *Saccharomyces pastorianus* yeast. J. Mass Spectrom..

[13] Richter T.M., Silcock P., Algarra A., Eyres G.T., Capozzi V., Bremer P.J., Biasioli F. (2018). Evaluation of PTR-ToF-MS as a tool to track the behavior of hop-derived compounds during the fermentation of beer. Food Res. Int..

[14] Buhr K., Van Ruth S., Delahunty C. (2002). Analysis of volatile flavour compounds by Proton Transfer Reaction-Mass Spectrometry: Fragmentation patterns and discrimination between isobaric and isomeric compounds. Int. J. Mass Spectrom..

[15] Tani A. (2013). Fragmentation and reaction rate constants of terpenoids determined by proton transfer reaction-mass spectrometry. Environ. Control Biol..

[16] Demarcke M., Amelynck C., Schoon N., Dhooghe F., Van Langenhove H., Dewulf J. (2009). Laboratory studies in support of the detection of sesquiterpenes by proton-transfer-reaction-mass-spectrometry. Int. J. Mass Spectrom..

[17] Maleknia S.D., Bell T.L., Adams M.A. (2007). PTR-MS analysis of reference and plant-emitted volatile organic compounds. Int. J. Mass Spectrom..

[18] Kim S., Karl T., Helmig D., Daly R., Rasmussen R., Guenther A. (2009). Measurement of atmospheric sesquiterpenes by proton transfer reaction-mass spectrometry (PTR-MS). Atmos. Meas. Tech..

[19] Aprea E., Biasioli F., Carlin S., Versini G., Märk T.D., Gasperi F. (2007). Rapid white truffle headspace analysis by proton transfer reaction mass spectrometry and comparison with solid-phase microextraction coupled with gas chromatography/mass spectrometry. Rapid Commun. Mass Spectrom..

[20] Lee A., Goldstein A.H., Kroll J.H., Ng N.L., Varutbangkul V., Flagan R.C., Seifeld J.H. (2006). Gas-phase products and secondary aerosol yields from the photooxidation of 16 different terpenes. J. Geophys. Res..

[21] Patrașcu L., Banu I., Bejan M., Aprodu I. (2018). Quality parameters of fruit beers available on Romanian market. Sci. Study Res..

[22] Maslanka R., Kwolek-Mirek M., Zadrag-Tecza R. (2018). Autofluorescence of yeast Saccharomyces cerevisiae cells caused by glucose metabolism products and its methodological implications. J. Microbiol. Methods.

[23] Taiti C., Stefano G., Percaccio E., Di Giacomo S., Iannone M., Marianelli A., Di Sotto A., Garzoli S. (2023). Addition of Spirulina to Craft Beer: Evaluation of the Effects on Volatile Flavor Profile and Cytoprotective Properties. Antioxidants.

[24] Sheraz M.A., Kazi S.H., Ahmed S., Anwar Z., Ahmad I. (2014). Photo, thermal and chemical degradation of riboflavin. Beilstein J. Org. Chem..

[25] Badalamenti N., Bruno M., Schicchi R., Geraci A., Leporini M., Gervasi L., Tundis R., Loizzo M.R. (2022). Chemical Compositions and Antioxidant Activities of Essential Oils, and Their Combinations, Obtained from Flavedo By-Product of Seven Cultivars of Sicilian *Citrus aurantium* L. Molecules.

[26] Shah B.B., Mehta A.A. (2018). In vitro evaluation of antioxidant activity of D-Limonene. Asian J. Pharm. Pharmacol..

[27] Nardini M. (2023). An Overview of Bioactive Phenolic Molecules and Antioxidant Properties of Beer: Emerging Trends. Molecules.

[28] De Keukeleire D., de Cooman L., Rong H., Heyerick A., Kalita J., Milligan S.R. (1999). Functional properties of hop polyphenols. Basic Life Sci..

[29] Nardini M., Garaguso I. (2020). Characterization of bioactive compounds and antioxidant activity of fruit beers. Food Chem..

[30] Pai T.V., Sawant S.Y., Ghatak A.A., Chaturvedi P.A., Gupte A.M., Desai N.S. (2015). Characterization of Indian beers: Chemical composition and antioxidant potential. J. Food Sci. Technol..

[31] Gouvinhas I., Breda C., Barros A.I. (2021). Characterization and Discrimination of Commercial Portuguese Beers Based on Phenolic Composition and Antioxidant Capacity. Foods.

[32] Nardini M., Foddai M.S. (2020). Phenolics Profile and Antioxidant Activity of Special Beers. Molecules.

[33] Lu Q., Huang N., Peng Y., Zhu C., Pan S. (2019). Peel oils from three *Citrus* species: Volatile constituents, antioxidant activities and related contributions of individual components. J. Food Sci. Technol..

[34] Di Sotto A., Durazzi F., Sarpietro M.G., Mazzanti G. (2013). Antimutagenic and antioxidant activities of some bioflavours from wine. Food Chem. Toxicol..

[35] Iannone M., Ovidi E., Vitalini S., Laghezza Masci V., Marianelli A., Iriti M., Tiezzi A., Garzoli S. (2022). From Hops to Craft Beers: Production Process, VOCs Profile Characterization, Total Polyphenol and Flavonoid Content Determination and Antioxidant Activity Evaluation. Processes.

[36] Ovidi E., Laghezza Masci V., Zambelli M., Tiezzi A., Vitalini S., Garzoli S. (2021). *Laurus nobilis*, *Salvia sclarea* and *Salvia officinalis* Essential Oils and Hydrolates: Evaluation of Liquid and Vapor Phase Chemical Composition and Biological Activities. Plants.

[37] Campbell-Sills H., Capozzi V., Romano A., Cappellin L., Spano G., Breniaux M., Biasioli F. (2016). Advances in wine analysis by PTR-ToF-MS: Optimization of the method and discrimination of wines from different geographical origins and fermented with different malolactic starters. Int. J. Mass Spectrom..

[38] Cappellin L., Biasioli F., Granitto P.M., Schuhfried E., Soukoulis C., Costa F., Märk T.D., Gasperi F. (2011). On data analysis in PTR-TOF-MS: From raw spectra to data mining. Sens. Actuators B Chem..

[39] Schindelin J., Arganda-Carreras I., Frise E., Kaynig V., Longair M., Pietzsch T., Preibisch S., Rueden C., Saalfeld S., Schmid B. (2012). Fiji: An open-source platform for biological-image analysis. Nat. Met..

[40] Vitalone A., Di Sotto A., Mammola C.L., Heyn R., Miglietta S., Mariani P., Sciubba F., Passarelli F., Nativio P., Mazzanti G. (2017). Phytochemical analysis and effects on ingestive behaviour of a *Caralluma fimbriata* extract. Food Chem. Toxicol..

[41] Di Giacomo S., Di Sotto A., Angelis A., Percaccio E., Vitalone A., Gullì M., Macone A., Axiotis E., Skaltsounis A.L. (2022). Phytochemical Composition and Cytoprotective Properties of the Endemic *Sideritis sipylea* Boiss Greek Species: A Valorization Study. Pharmaceuticals.

[42] Di Sotto A., Locatelli M., Macone A., Toniolo C., Cesa S., Carradori S., Eufemi M., Mazzanti G., Di Giacomo S. (2019). Hypoglycemic, antiglycation, and cytoprotective properties of a phenol-rich extract from waste peel of *Punica granatum* L. var. Dente di Cavallo DC2. Molecules.

[43] Nikfarjam L., Farzaneh P. (2012). Prevention and detection of Mycoplasma contamination in cell culture. Cell J..

[44] European Pharmacopeia (2002). Biological Tests-Mycoplasmas.

[45] (2009). Biological Evaluation of Medical Devices Part 5: Tests for In Vitro Cytotoxicity.

